# Ligation-assisted endoscopic mucosal resection has high complete resection rate in rectal carcinoid tumor

**DOI:** 10.1186/s12876-021-02061-4

**Published:** 2021-12-13

**Authors:** Ming-Yao Su, Cheng-Tang Chiu

**Affiliations:** 1 Department of Internal Medicine, New Taipei Municipal TuCheng Hospital, 6, Sec 2, Jincheng Rd, Tucheng Dist, New Taipei City, Taiwan, Republic of China; 2 Department of Gastroenterology and Hepatology, Chang Gung Memorial Hospital Linkou Branch, Taoyuan, Taiwan; 3 Chang Gung University College of Medicine, Taoyuan, Taiwan; 4 Taiwan Association for the Study of Small Intestinal Diseases, Taoyuan, Taiwan

**Keywords:** Carcinoid, Neuroendocrine tumor, Endoscopic mucosal resection, Ligation

## Abstract

**Aim:**

We aimed to compare the outcomes of different therapeutic modalities in rectal carcinoid tumors.

**Method:**

We retrospectively collected 145 patients with rectal carcinoid tumors which were pathologically diagnosed from 2005/01/01 to 2016/12/31. We compared tumor size, complete resection rate and recurrent rate between different therapeutic modalities. Then, prospectively compared the treatment outcomes of 28 patients treated with ligation assisted endoscopic mucosal resection (LEMR) and 25 patients treated with endoscopic mucosal resection with cap (EMRC).

**Result:**

The mean size of tumors was 6.5 mm (1–25 mm), and the mean follow-up duration was 26 months (6–118 months). The therapeutic modalities included ligation-assisted endoscopic mucosal resection (LEMR) (25 tumors, 17%), endoscopic mucosal resection (EMR) (31 tumors, 21%), snare polypectomy (30 tumors, 21%), biopsy forceps removal (46 tumors, 32%) and surgical resection (13 tumors, 11%), including 6 tumors treated with transanal endoscopic microsurgery (TEM) method. In view of pathologically complete resection rate, LEMR was highest (100%), followed by surgical resection (85%). However, EMR only had 42% pathologically complete resection rate. Besides, LEMR and surgical resection had no local recurrence and significantly higher clinically complete resection rate, compared to other treatments. For the further prospective study, complete resection was noted in 28 (100%) patients in LEMR group and 13 (52%) patients in EMRC group.

**Conclusion:**

In the treatment of rectal carcinoid tumors, LEMR is safe and effective compared with traditional endoscopic treatments.

## Introduction

Rectal carcinoid tumors are usually found incidentally during endoscopic examination. The typically endoscopic appearance of rectal carcinoid tumors is smooth, round, sessile elevations covered with normal-appearing or yellow-discolored mucosa [1]. The clinical characteristics of rectal carcinoid tumors include male predominance (1.6:1), small-sized tumors of 10 mm or less in detection (66.0%), infrequent association with carcinoid syndrome (0.7%), and relatively high 5-year survival rate after removal (81.5%) [2].

We can evaluate the malignant potential of rectal carcinoid tumors according to tumor size, endoscopic features, histological growth patterns, muscularis propria invasion, and lymphovascular invasion [1–3]. Among these parameters, primary tumor size is most simple and reliable predictor. Metastatic rate is less than 3% in tumors ≦ 10 mm in diameter and 5–15% in tumors between 11 and 20 mm in diameter. However, it is up to 80% in the tumor > 20 mm [4–6]. Previous studies suggested local resection for the carcinoid tumors less than 10 mm and confined to the submucosa because of low metastatic rate [7].

No matter endoscopic or surgical treatments, there were several methods for rectal carcinoid tumor resection. However, the therapeutic outcomes between different modalities are still unknown, and we aim to clarify the issue.

## Patients and methods

### Endoscopic procedures

#### Endoscopic mucosal resection (EMR)

After injecting saline solution beneath the tumor to lift it away from the muscularis propria, snare resection by serrated snare 30 mm in size with blended electrosurgical current was performed. For the retrospective analysis, the lesions were hot polypectomized or EMR, which were differentiated by submucosal fluid injection or no and used smooth snare or serrated snare. The EMR method had submucosal fluid injection combined with serrated snare.

#### Endoscopic mucosal resection with cap (EMRC)

Saline solution was injected into submucosal layer beneath the tumor to reduce the risks of perforation and resection margin involvement. The tumor was then aspirated into the cap, followed by snaring with the snare attached within the tip of cap. The snare resection was finally performed with blended electrosurgical current.

#### Ligation-assisted endoscopic mucosal resection (LEMR)

**Fig. 1:**
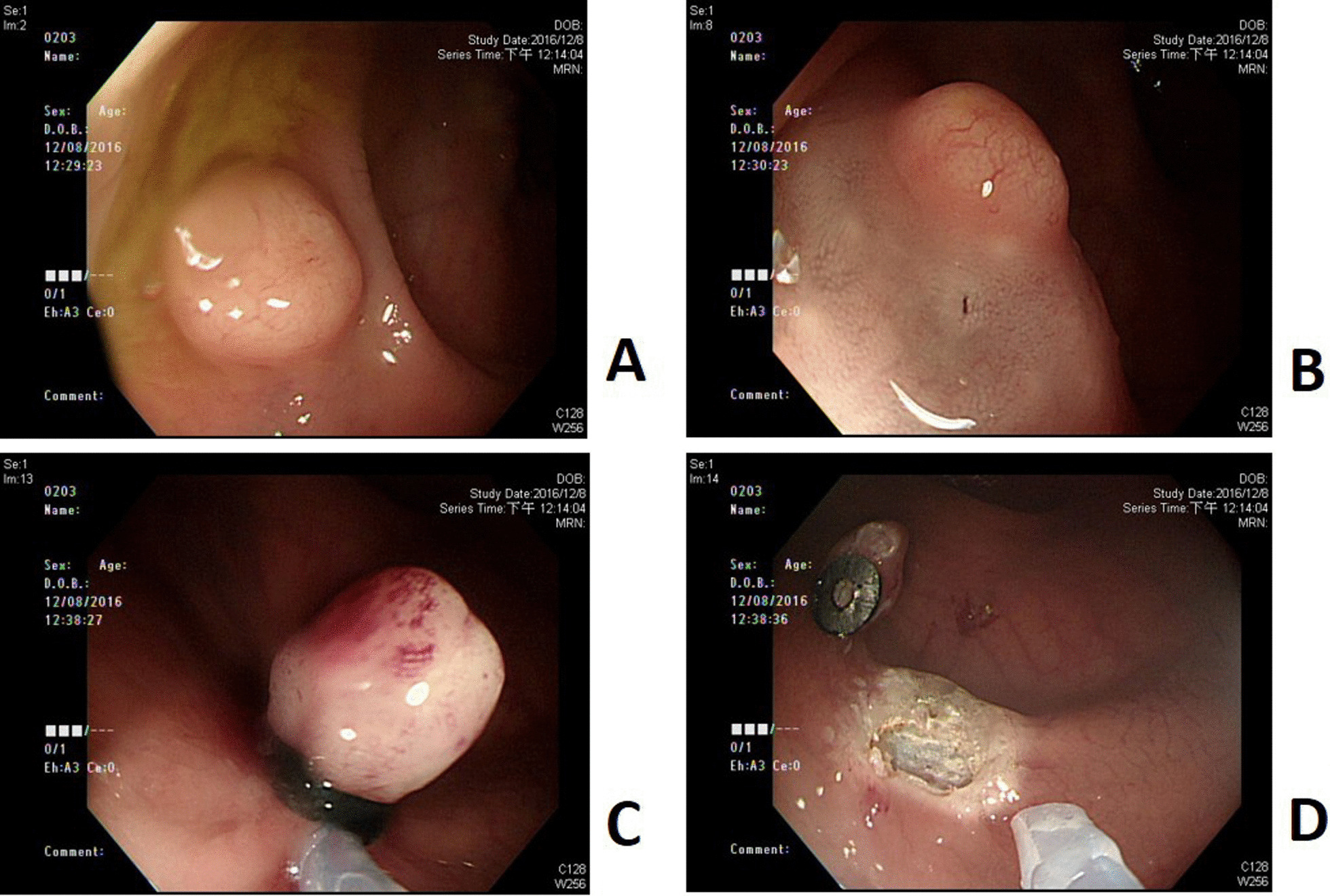
The endoscopic procedure of LEMR. **A** Identify the lesion by endoscopy. **B** Submucosal saline solution was injected beneath the tumor to elevate it. **C** The lesion was then aspirated into the ligation device, followed by deployment of the elastic band. **D** Snare resection was performed below the band by using blended electrosurgical current

The procedure was carried out with a conventional single-channel endoscope (GIF-260, GIF-290, Olympus) with multi-band ligator device (Cook). It was then performed by following steps: (1) After identifying the lesion (Fig. 1A), we did submucosal saline solution injection beneath the tumor (Fig. 1B); (2) The tumor was aspirated into the ligation device, followed by deployment of the elastic band (Fig. 1C); (3) Snare resection with blended electrosurgical current was performed below the ligation band (Fig. 1D).

### Histopathology evaluation

After cutting the specimens into 2 mm slices, H&E staining was performed to obverse the histopathology type, horizontal resected margin and vertical resected margin. The involvement of resection margin was decided according to the distance between tumor border and resection margin.

### Patients

During 2005/01/01 to 2016/12/31, we retrospectively collected 145 rectal carcinoid tumor patients with pathological diagnosis in Chang Gung Memorial Hospital Linkou branch. All the patients had signed for the informed consent and this study was approved by the Chang Gung Medical Foundation Institutional Review Board number 201502259B0C501, and all methods were performed in accordance with the relevant guidelines and regulations. We exclude 31 patients who lost follow-up for the evaluation of recurrence. All enrolled patients had no clinical symptoms or signs of Carcinoid syndrome and tumors were found incidentally by colonoscopy check-up. The size of rectal carcinoid tumors and the methods of treatment were recorded. Therapeutic modalities were determined by the colonoscopists and proctologists during endoscopic examination. The primary outcomes included complete resection rate and local recurrent rate. Besides, the clinically complete resection rate (no recurrence found during follow-up) was also reviewed. All the polypectomy or EMR treatment were used hot coagulations, no cold snare used in this study. And all the procedures were performed by one experienced colonoscopist, who had performed more than 50,000 colonoscopies, 15,000 polypectomies and 2000 EMR procedures. Because the carcinoid tumors were subepithelial lesions, some of the resected margin of specimen were causative injury by coagulation and these cases were included with incomplete resection.

According to retrospective data, LEMR was 100% (25/25) and ENR was 42% (13/31) complete resection rates, we conducted a prospective comparison of these two methods which need 25 patients each arm at least. We didn’t consider of local recurrent rate for the primary end point. The patients were receiving EMR or LEMR therapy by randomized selection. There were 53 consecutive patients with rectal carcinoid tumors were randomized treated with LEMR (28 patients) and EMRC (25 patients) from 2017/1/1 to 2019/6/30. The complete resection rates were compared by this two groups of patients.

All data generated or analyzed during this study are included in this article. Please contacted Dr Ming-Yao Su if someone wants to request the data.

## Result

The characteristics of these tumors from 145 patients are listed in Table 1. There were 57 males (39%) and 88 females (61%). The mean age at diagnosis was 49 years (range, 21–77 years). The mean size of tumor was 6.5 mm (range, 1–25 mm) with average distance to anus about 6 cm. Patients continued to undergo follow-up colonoscopy or sigmoidoscopy annually. The mean follow-up period was 26 months (range 6–118 months).

**Table 1:** The characteristics of patients and tumors

	LEMR	EMR	Surgery	Polypectomy	Biopsy
Patient number	25	31	13	30	46
Age	51 ± 16	49 ± 19	43 ± 21	58 ± 15	44 ± 21
Male (%)	40	39	46	37	39
Tumor size (mm)	6 ± 3	5 ± 2	7 ± 5	5 ± 4	6 ± 4
BMI (mean)	23.6	22.9	24.1	22.3	22.5
Average distance to anus (cm)	6.7	6.3	6.5	5.8	6

Thirty-one (21%) tumors were treated with EMR, including 16 tumors were treated with EMR with cap (EMRC). Thirty (21%) tumors were treated with snare polypectomy. Forty-six (32%) tumors were treated with biopsy forceps removal. Thirteen (9%) tumors were treated with surgical resection, including 6 tumors were treated with transanal endoscopic microsurgery (TEM) method. Twenty-five (17%) tumors were treated with LEMR.

All the pathologic diagnoses were neuroendocrine tumor grade 1 with low mitotic rate (< 5 in one high power view). No muscularis propria or lymphovascular invasion found in all complete resected specimens.

The treatment outcomes of rectal carcinoid tumors are listed in Table 2. The complete endoscopic resection rates were 100% for LEMR, 97% for EMR and 60% for polypectomy. According to pathologic diagnosis, the LEMR group has the highest complete resection rate (100%) based on pathologic evaluation, followed by surgical groups (85%). The EMR group has about half complete resection rate (42%) pathologically. While the polypectomy and biopsy groups had lowest complete resection rate based on pathologic evaluation (3 and 4%). The average resected margins for the complete resected tumors in each groups were 1.3 mm in LEMR group, 1.5 mm in surgical group, 0.4 mm in EMR group, 0.5 mm in polypectomy group and 0.1 mm in biopsy group, there significant difference between LEMR and surgical group to other groups (*p* < 0.05). The 2 patients which had inadequate resection in surgical group were their lesions were too close to anus, so the surgeon consider to preserve the anal function so the resected margin were very close to tumor margin. Also, the clinically complete resection rate was high in the surgical resection and LEMR group (100%). The local recurrent rate was higher in other groups (29% in EMR group, 83% in polypectomy group and 93% in biopsy group), as Table 2. No major complications such as major bleeding or perforation occurred in current study. No metastatic lesion found during the period of follow- up. Except the surgical group, all patients received the endoscopic procedure at out-patient department.

**Table 2:** Treatment outcomes of rectal carcinoid tumors

	Number	Tumor size	CR-E	CR-P	ARM (mm)	ICR-P	UR-P	CR-C	Follow up period (months)	Recurrence rate (%)
LEMR	25 (17%)	6.3 ± 2.7 mm (range, 3–11 mm)	25 (100%)	25 (100%)	1.3	0 (0%)	0 (0%)	100% (25/25)	8.6 (6–26)	0
EMR	31 (21%)	6.6 ± 2.3 mm (range, 3–12 mm)	30 (97%)	13 (42%)	0.4	8 (26%)	10 (32%)	71% (22/31)	12.8 (8–61)	29
Polypectomy	30 (21%)	7.4 ± 4.5 mm (range, 2–20 mm)	18 (60%)	1 (3%)	0.5	17 (57%)	12 (40%)	17% (3/18)	19.3 (2–47)	83
Biopsy removal	46 (32%)	3.4 ± 1.5 mm (range, 1–6 mm)		2 (4%)	0.1	21 (46%)	23 (50%)	7% (2/27)	21.6 (1–84)	93
Surgical resection	13 (9%)	9.7 ± 7.8 mm (range, 2–25 mm)		11 (85%)	1.5	0 (0%)	2 (15%)	100% (13/13)	27.0 (9–91)	0

CR-E, complete resection based on endoscopic evaluation; CR-P, complete resection based on pathologic evaluation; ICR-P, incomplete resection based on pathologic evaluation; UR-P, undetermined resection margin based on pathologic evaluation; CR-C, complete resection based on clinical follow up; ARM, average resection margin for complete resected tumors

We further compared the complete resection rate between LEMR and EMRC groups. 53 rectal carcinoid tumors were found incidentally by routine colonoscopy from 2017/1/1 to 2019/6/30 were randomized treated with LEMR 928 patients) and EMRC 925 patients) prospectively. No differences noted for the patients’ characteristics included sex, age and tumor size for this two groups of patients (Table 3). However, the complete resection rate was 100% in LEMR group and 52% in EMRC group (*p* < 0.005).

**Table 3:** The characteristics of patients treated with LEMR and EMR

	LEMR	EMR	*p* value
Patient number	28	25	NS
Age	48 ± 12	50 ± 14	NS
Male (%)	50	52	NS
Tumor size (mm)	6 ± 4	5 ± 3	NS
BMI (mean)	24.2	23.9	NS
Average distance to anus (cm)	6.2	6.4	NS
Complete resection rate (%)	100	52	*p* < 0.005

## Discussion

Several features have been reported as possible predictors for outcomes in patient with rectal carcinoid tumors. The features associated with a poor prognosis include large size, deep invasion, lymphovascular invasion, and elevated mitotic rate [8, 9]. However, the size of the primary tumor was a simple and reliable factor for predicting the risk of metastasis. Therefore, rectal carcinoid tumors are considered as a good candidate for local excision, including endoscopic or transanal resection. When the tumor was less than 10 mm in diameter, without atypical features and confined to the submucosal layer, the possibility of lymphovascular invasion or distant metastasis was very rare [10–12]. Various methods of endoscopic resection for rectal carcinoid tumors have been developed. Endoscopic mucosal resection with or without cap is considered as an effective method for complete resection.

During conventional endoscopic mucosal resection (EMR), the lesions were elevated by injecting saline (or other solute) into the underlying submucosal layer and then snared and resected using a blend current [13]. Due to the submucosal nature of rectal carcinoid tumors and sharpness of snare, conventional EMR is more likely to associated with incomplete resection margins [14, 15]. Another alternative to conventional EMR involves suctioning the area raised by solute injection into a transparent cap (EMR-C) and either cleaving the lesion directly or banding it, with subsequent snare resection and retrieval. Some pilot studies suggest that these methods may be more effective [16–25]. However, most of these studies only enrolled limited number of cases. In our series, the complete histopathologically resection rate by EMR or EMRC method is only 42%. The reason may be related to the characteristic of snare, which is very thin and sharp, and would cut through the tumor and resulted in incomplete resection. The recurrence lesions were found as soon as one month after local management, so, we suggest f/u colonoscopy at least one month later after incomplete resection procedures.

During ligation-assisted endoscopic mucosal resection (LEMR), a rubber band was used to ligate the tumor before the procedure. Because the rubber band can follow the curve of tumor, it would be tight below the lesion strongly and facilitate further en-bloc snaring and resection. In our study, by this LEMR method, all lesions can be resected completely by histopathology evaluation with adequate resected margin.

Complete resection of carcinoid tumors of the rectum remains be difficult by conventional polypectomy or biopsy method. In our study, the rate of complete resection was only 3–4%, which is inferior to the results obtained by EMR, EMRC or LEMR. According to our current study, we suggest that biopsy removal or polypectomy are not adequate treatment method for rectal submucosal lesion. And the lesion should be resected by other advanced procedures, such as LEMR or surgery, to achieve high complete resection rate. The 2 patients which had inadequate resection in surgical group were their lesions were too close to anus, so the surgeon consider to preserve the anal function so the resected margin were very close to tumor margin, so for the lesions close to anus may choose LEMR for the treatment option.

Although the complete resection rate was not high for EMR or EMRC, the local recurrence was not high in our study. Possible reasons for this observation include: (1) electrocoagulation may have caused necrosis of the peripheral margins of the resected specimens; (2) the behavior of these carcinoid tumors was indolent; and (3) the follow-up period was too short for tumor recurrences.

Further prospectively analysis of complete resection rates compared with LEMR and EMRC groups, which showed that significant difference between this two groups (100% in LEMR and 52% in EMRC group, *p* < 0.005). Similar result was shown by our previous retrospective analysis.

For some lesions more than 2 cm in size, LEMR may be not suitable for the limitation of cap’s size, while endoscopic mucosal dissection (ESD) may be an alternative treatment, but ESD need more learning curve and we will start another study for the treatment outcomes of ESD for rectal carcinoid tumors.

In the past, all recurrent cases were refer to surgery for further management; however, after the retrospective analysis data noted, we treated the later cases by LEMR with good results (100% complete resection rate).

In conclusion, for rectal carcinoid tumors, LEMR had highest complete resection rate with adequate resected margin and no local recurrence during follow-up even compared with EMR. Although surgery also had high complete resection rate and no local recurrence found, it takes the cost of admission and the risk of anesthesia. LEMR is a safe and effective modality for treating rectal carcinoid tumors.

## Data Availability

Please contacted Dr Ming-Yao Su if someone wants to request the data.
